# Resistance of glioma cells to nutrient-deprived microenvironment can be enhanced by CD133-mediated autophagy

**DOI:** 10.18632/oncotarget.12803

**Published:** 2016-10-21

**Authors:** Haojie Sun, Mingzhi Zhang, Kai Cheng, Peng Li, Shuo Han, Ruizhi Li, Ming Su, Wotan Zeng, Jinwen Liu, Jinhai Guo, Yinan Liu, Xiaoyan Zhang, Qihua He, Li Shen

**Affiliations:** ^1^ Department of Cell Biology, Stem Cell Research Center, School of Basic Medical Sciences, Peking University Health Science Center, Beijing, People's Republic of China; ^2^ Department of Laboratory Medicine, Fenyang College of Shanxi Medical University, Fenyang, People's Republic of China; ^3^ Beijing DongFang YaMei Gene Science and Technology Research Institute, Beijing, People's Republic of China

**Keywords:** CD133, autophagy, mTOR, Beclin1, Atg5

## Abstract

CD133 is a pentaspan transmembrane protein that can serve as a biomarker for cancer stem cells, although its biochemical mechanism remains unclear. Here we report that CD133 expression enhances glioma cell tolerance of a nutrient-deprived microenvironment. Under starvation conditions, CD133-positive cells exhibited higher survival and decreased levels of apoptosis. These changes were dependent on activation of autophagy-associated gene signaling and were impaired by the autophagic inhibitor chloroquine. Furthermore, rapamycin up-regulated the level of autophagy and inversely reduced CD133 expression. Immunofluorescence confirmed that starvation promoted release of CD133 from the plasma membrane to the cytoplasm, with CD133 also partially co-localizing with LC3 upon starvation. Additionally, CD133 partially co-localized with Beclin1, Atg5, and lysosomes, indicating that CD133 directly participates in the autophagosome membrane fusion process and ultimately undergoes lysosomal degradation. Collectively, our results demonstrate that CD133 contributes to cell survival by regulating autophagy, and that targeting CD133-linked signaling and autophagy may be useful in improving anti-cancer treatments.

## INTRODUCTION

Glioma is the leading cause of central nervous system-derived cancer-related death [1]. Presently, the molecular mechanisms underlying glioma development remain incompletely understood. The identification of a minority subpopulation of cells that are in charge of tumorigenesis and tumor maintenance in breast cancer and leukemia led to the proposition of the cancer stem cell (CSC) theory [2]. This theory holds that CSCs are a minority population within the cancer that have the ability to selfrenew and proliferate extensively to maintain tumor growth [3]. A small cohort of CSCs in multiple glioma types have been successfully separated by some research groups [4–6]. These cells exhibit extremely aggressive biological behavior and are essential for cancer formation, growth, and recurrence. The cell surface protein CD133 is a biomarker used to identify several types of stem cells [7, 8]. Singh et al. isolated CD133^+^ glioblastoma CSCs and found that these cells developed xenographic tumors while CD133^−^ cells did not [8]. CD133^+^ stem cells have also been found in osteosarcoma, hepatocarcinoma, melanoma, breast cancer, and colorectal cancer [8–13]. Therefore, CD133 could represent a valuable biomarker of CSCs.

CD133 is the product of a single-copy gene on chromosome 5 (5b3) in mice and chromosome 4 (4p15.33) in humans [14]. CD133 interacts with cholesterol and selectively enriches in plasma membrane protrusions [15–17]. CD133 deficiency leads to disk dysmorphogenesis and photoreceptor degeneration [18]. Meanwhile, CD133-positive membrane particles have been identified in various body fluids from human adults, including urine, saliva, and seminal fluid [19, 20]. CD133 is also related to alterations of mitochondrial function and cholesterol metabolism in glioma [21, 22]. Clarifying the functions of CD133 in cancer and incorporating these discoveries into drug application may be helpful for cancer therapy [23].

The tumor microenvironment must also be considered in the pathogenesis and progression of gliomas [24]. Hypoxia and ischemia may be inevitable outcomes of a rapidly-growing tumor outstripping its vascular supply [25], with hypoxia and ischemia frequently leading to autophagy. Autophagy is an evolutionarily conserved pathway that benefits cells via lysosomal-mediated degradation of aggregate-prone proteins and damaged organelles during times of stress [26, 27]. CD133 is released from the plasma membrane to the cytoplasm in hepatoma cells, and CD133 expression promotes glucose uptake under conditions of glucose deprivation [28]. Autophagy induced by gamma irradiation confers a tolerance upon CD133-positive glioma cells that can be inhibited by chloroquine [29]. These findings indicate that CD133 regulates autophagy, but the exact molecular mechanism remains elusive.

In this study, we found that CD133 can translocate from the plasma membrane to the cytoplasm in glioma, thereby enhancing resistance to a nutrient-deprived microenvironment. Furthermore, we report that CD133 participates in autophagy to promote resistance to nutritional starvation in glioma cells.

## RESULTS

### CD133 improves the resistance of glioma cells to a nutrient-deprived microenvironment

**Figure 1:**
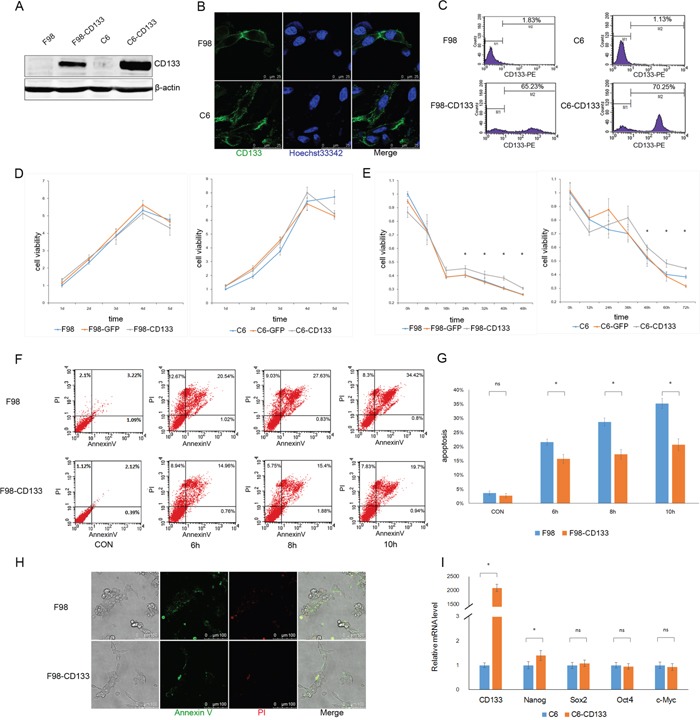
CD133^+^ cells exhibit lower sensitivity to nutrient-deprived microenvironment compared to CD133^−^ cells **A.** Recombinant lentiviruses containing CD133 were transduced into F98 and C6 rat glioma cells, level of CD133 protein was determined by Western blotting after one week puromycin selection. **B** and **C.** Expression of CD133 was evaluated by immunofluorescence microscopy (B) and flow cytometry (C). **D.** F98/C6, F98/C6-GFP and F98/C6-CD133 cells were maintained under normal culture medium, cell viability was detected by CCK8 at 1, 2, 3, 4 and 5 d and the folds of proliferation were obtained by the ratios of the value of each time over the one at initial point respectively. **E.** F98/C6, F98/C6-GFP and F98/C6-CD133 cells were cultured under glucose free medium, cell viability was detected by CCK8 at the indicated time. Statistical analysis was done as shown in (D). *P<0.05. **F.** F98 and F98-CD133 cells were treated with EBSS for 6, 8 and 10h to determine the apoptosis and necrosis. The percentage of apoptosis and necrosis was analyzed by Annexin V-FITC/PI double staining via flow cytometry after the designated treatments. **G.** Percentage of Annexin V positive cells was quantified. *P<0.05. **H.** The percentage of apoptosis and necrosis was evaluated by Annexin V-FITC/PI double staining via immunofluorescence microscopy after cells exposed to EBSS for 10h. **I.** Real-time PCR analysis of stem cell-associated genes in F98/F98-CD133 cells. Relative gene expression to F98 cells was calculated for F98-CD133 cells and presented in the bar graphs with standard deviations. *P<0.05.

We investigated the biofunction of CD133 in glioma cells by establishing F98-CD133 and C6-CD133 cell lines that stably-expressed CD133 via lentivirus infection (Figure 1A–1C). We evaluated the proliferation rates of F98/F98-CD133 and C6/C6-CD133 cells in complete medium to determine whether CD133 influences cell proliferation and survival. Figure 1D demonstrates that CD133 did not influence cell proliferation under normal culture conditions. Given that glioma cells are subjected to nutrition deprivation *in vivo* [25], glucose was removed from the culture medium to replicate a nutrient-deficient microenvironment. CD133^+^ cells cultured in glucose-free medium exhibited significant higher cell viability when compared with CD133^−^ cells (Figure 1E). Meanwhile, CD133^+^ cells showed lower levels of apoptosis and necrosis when treated with Earle's Balanced Salt Solution (EBSS) (Figure 1F–1H, Supplemental Figure S1A–S1C). Next, we investigated whether transfection of CD133 could enhance the stemness of glioma cells. Figure 1I shows that CD133 overexpression produced slight increases in the expression levels of stemness-associated transcription factors in F98 cells. Taken together, these findings indicate that CD133 is helpful for cell survival in a nutrient-deprived microenvironment.

### Altered levels of autophagy account for the lower sensitivity of CD133^+^ cells to a nutrient-depleted microenvironment

**Figure 2:**
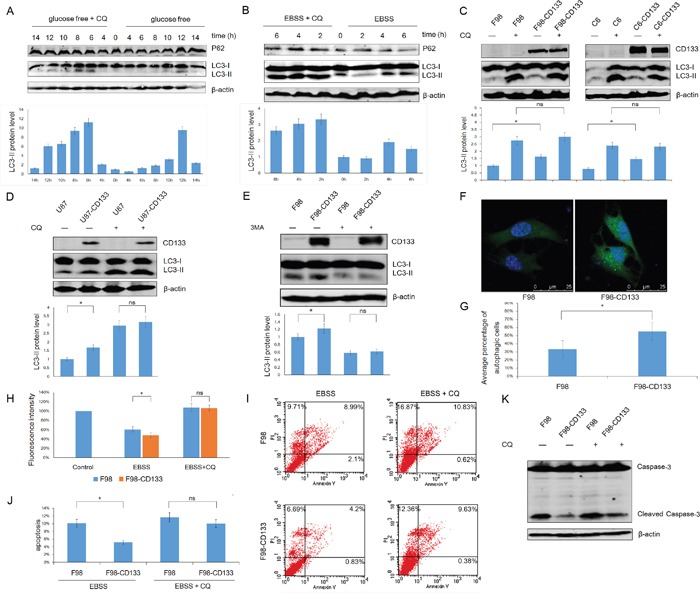
Autophagy confers on CD133^+^ cells the tolerance to stress microenvironment **A.** F98 cells were cultured under glucose free medium for indicated time with or without 50uM CQ, expression of LC3-II and P62 was evaluated by Western blot analysis. The intensity of bands was determined with the image J program and the folds of intensity were obtained by the ratios of the value of each group over control respectively and shown in graphs under immunoblots. Data of three replicates were shown as means ± SD. **B.** F98 cells were treated with EBSS for indicated time in presence or absence of 50uM CQ, lysates prepared from the designated treatment groups were subjected to Western blot analysis with the indicated antibodies. Densitometric analysis was done as described in (A). **C** and **D.** Levels of LC3-II in these indicated cell lines were evaluated by Western blot after incubating in EBSS for 4h with or without 50uM CQ. Densitometric analysis was carried out as described in (A). *P<0.05. **E.** Levels of LC3-II in F98/F98-CD133 cell lines were evaluated by Western blot after incubating in EBSS for 4h with or without 5mM 3-MA. Densitometric analysis was done as described in (A). *P<0.05. **F.** F98 cells were transfected with GFP-tagged LC3. 24 h after transfection, cells were incubated with BESS for 4h, the representative pictures were selected from observation. **G.** The percentage of cells with punctate GFP-LC3 fluorescence was evaluated. Cells with more than six autophagosomes per cell were scored as autophagic positive cells. *P<0.05. **H.** Graph presentation. FACS analysis of F98/F98-CD133 cells transiently expressing wild-type GFP-LC3 incubated in complete medium (control), EBSS, or EBSS containing 50uM CQ for 6h. Data of three replicates were shown as means ± SD. *P<0.05. **I.** F98 and F98-CD133 cells were treated with EBSS for 4h in presence or absence of 50uM CQ. The percentage of apoptosis and necrosis was analyzed by Annexin V-FITC/PI double staining via flow cytometry. **J.** Percentage of Annexin V positive cells was quantified. *P<0.05. **K.** Activation of Caspase-3 in F98 and F98-CD133 cells was examined by Western blot after incubating in EBSS for 8h with or without 50uM CQ.

Low levels of glucose and nutritional deprivation trigger autophagic activity. We next used F98 cells to examine whether autophagy by CD133^+^ cells facilitates tolerance to a stressful microenvironment. Figure 2A and 2B show that exposure of F98 cells to EBSS and glucose-free medium upregulated levels of autophagy in a time-dependent manner, and that autophagy could be inhibited by CQ. Furthermore, CD133^+^ cells expressed a higher LC3-II level under EBSS culture conditions and this phenomenon could be abrogated by the autophagy inhibitor CQ (Figure 2C and 2D, Supplemental Figure S2A and S2B). Additionally, treatment of F98/F98-CD133 cells with the autophagy inhibitor 3-MA inhibited CD133-induced autophagy (Figure 2E). To identify the activation of autophagy in glioma cells, F98/F98-CD133 cells were transiently transfected with the GFP-LC3 vector. Activation of autophagy promotes ubiquitin-like conjugation of LC3 to PE, and these conjugates concentrate in autophagic vacuoles to form punctate structures [31]. More GFP-LC3 foci were present in CD133 positive cells compared with control cells after treatment by EBSS (Figures 2F and 2G). Following conjugation to PE, a significant portion of GFP-LC3 is delivered to lysosomes for degradation, resulting in reduced GFP fluorescence intensity in starved cells. Therefore, flow cytometry can be used to quantify the turnover of GFP-LC3, a read out of effective autophagy in mammalian cells [32]. F98-CD133 cells exhibited lower levels of GFP-LC3 fluorescence, while CQ inhibited the decay of GFP-LC3 fluorescence intensity (Figure 2H). Annexin V-FITC and PI staining showed that levels of apoptosis and necrosis increased when cells were treated with EBSS after inhibition of autophagy by CQ. Additionally, the different levels of apoptotic and necrotic cells between CD133 positive and CD133 negative subpopulations were attenuated after autophagy inhibition (Figure 2I and 2J). Meanwhile, caspase-3 activation in F98-CD133 cells was less than that in control cells following EBSS treatment (Figure 2K). These findings indicate that autophagy is critically essential for CD133^+^ cells survival by promoting resistance to EBSS-induced apoptosis and necrosis.

### Activation of autophagy-associated signaling pathways by CD133 confers tolerance to nutritional starvation upon glioma cells

**Figure 3:**
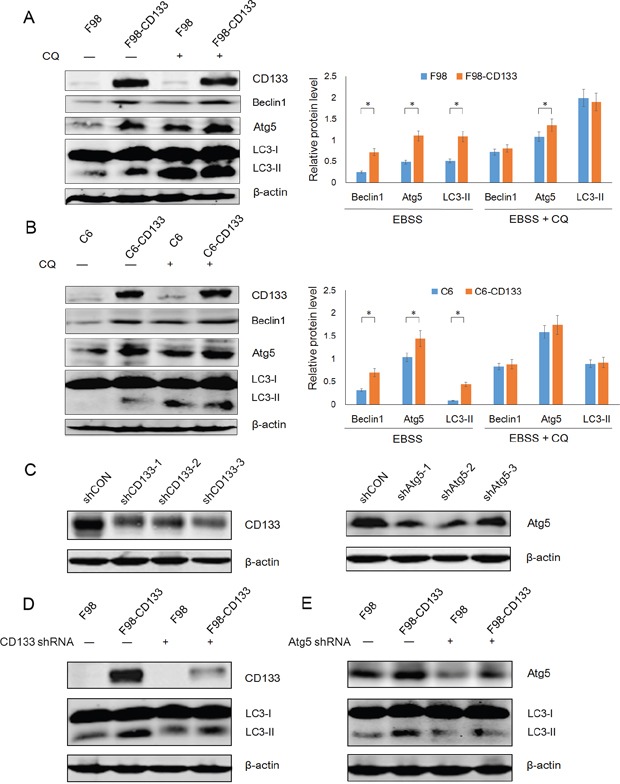
CD133-mediated autophagy is directly dependent on autophagy-associated genes **A.** Detection of autophagic genes in F98 and F98-CD133 Cells by Western blotting. Cells were harvested at 4h after culturing in EBSS with or without 50uM CQ. Densitometric analysis was done by normalization of autophagy-associated proteins levels with their own β-actin and shown under Western blotting panels. *P<0.05. **B.** Detection of autophagic genes in C6 and C6-CD133 Cells by Western blotting. Cells were treated as described in (A). Densitometric analysis was carried out as shown in (A). *P<0.05. **C.** F98-CD133 cells were transfected with several shRNA respectively for 24h, immunoblotting for CD133 and Atg5 confirmed knockdown by shRNA. **D.** F98/F98-CD133 cells were treated with EBSS for 4h after CD133 shRNA treatment for 24h, expression of CD133 and LC3-II was evaluated by Western blot analysis. **E.** F98/F98-CD133 cells were treated with EBSS for 4h after Atg5 shRNA treatment for 24h, expression of Atg5 and LC3-II was evaluated by Western blot analysis.

Autophagy is an evolutionarily conserved catabolic process for the degradation of damaged proteins and organelles via lysosome, and its activation in response to nutrient deprivation is regulated by PI3K/mTOR pathway [33]. We next investigated the mechanism by which CD133 promotes cell survival under conditions of nutritional starvation by examining whether CD133 could regulate autophagy or autophagy-associated genes. Western blotting showed that CD133 could up-regulate the expression of Beclin1 and Atg5 when cells were treated with EBSS. However, the differences in levels of Beclin1 and Atg5 expression between CD133 positive and CD133 negative cells were attenuated after autophagy inhibition (Figure 3A and 3B). Additionally, we produced three shRNA constructs targeting each of CD133 and Atg5 respectively. Figure 3C demonstrates that shCD133-3 and shAtg5-2 effectively inhibited the expression of CD133 and Atg5, respectively. These constructs were used for subsequent experiments. The different levels of autophagic activity between CD133^+^ and CD133^−^ subpopulations were attenuated when expression of CD133 was downregulated by shRNA (Figure 3D). Meanwhile, shAtg5 significantly inhibited autophagy in both F98 and F98-CD133 cells (Figure 3E). These results suggest that CD133-mediated autophagy directly depends on alterations to the expression of autophagy-associated signaling pathways.

### CD133 and CD133-mediated autophagy are regulated by mTOR signaling

**Figure 4:**
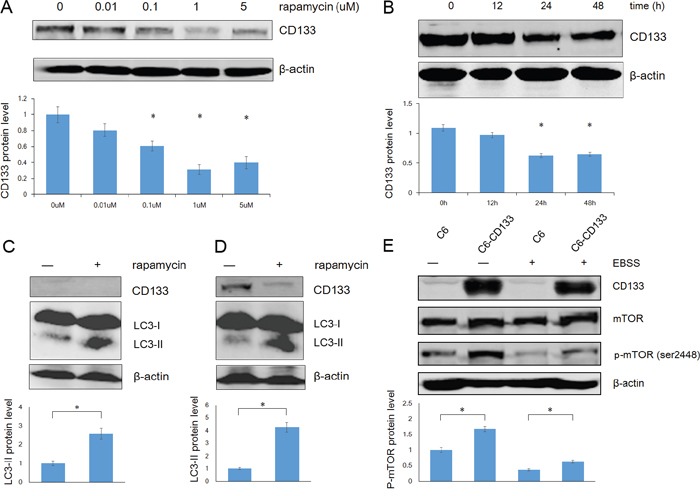
Rapamycin down-regulates CD133 expression and up-regulates LC3-II expression in glioma cells **A.** The dose curve of rapamycin in treatment of C6 glioma cells in concentrations of 0.01, 0.1, 1 and 5uM for 48h. The intensity of bands was determined with the image J program and the folds of intensity were obtained by the ratios of the value of each group over control respectively. Data of three replicates were shown as means ± SD. *P < 0.05. **B.** The time course of rapamycin in treatment of C6 glioma cells in a concentration of 1uM. The intensity was analysed as described in (A). **C.** C6 cells were exposed to EBSS for 4h with or without 1uM rapamycin, expression of CD133 and LC3 was evaluated by Western blot analysis. The intensity was carried out as described in (A). *P < 0.05. **D.** C6-CD133 cells were treated as described in (C), levels of CD133 and LC3 proteins were determined by Western blot analysis. Densitometric analysis was done as shown in (A). *P<0.05. **E.** C6/C6-CD133 cells were cultured under complete medium (control) or EBSS for 4h, immunoblotting for mTOR and p-mTOR (ser2448). Densitometric analysis was carried out as described in (A). *P<0.05.

Autophagy is regulated by the PI3K/mTOR signaling pathway. Kazuko et al. found that inhibition of mTOR signaling up-regulates CD133 expression in gastric and colorectal cancer cells [34]. Contrastingly, we found that rapamycin down-regulated CD133 expression in time- and dose-dependent manners in C6 glioma cells (Figure 4A and 4B). We further evaluated the relationships among CD133, autophagy, and mTOR signaling by measuring LC3 and CD133 protein levels after exposing cells to EBSS for 4 h with or without 1 μM rapamycin. Rapamycin reduced the expression of CD133 and inversely up-regulated LC3-II (Figure 4C and 4D). Furthermore, C6-CD133 cells exhibited higher activation of mTOR compared with C6 cells, and nutrient deprivation inactivated mTOR in both C6 and C6-CD133 cells (Figure 4E). Therefore, CD133 may regulate a parallel signaling pathway that exhibits crosstalk with mTOR signaling and ultimately regulate autophagy-associated gene expression, the influence of CD133 on autophagy can be covered by mTOR signal.

### CD133 directly participates in the membrane fusion processes required for autophagosome biogenesis

**Figure 5:**
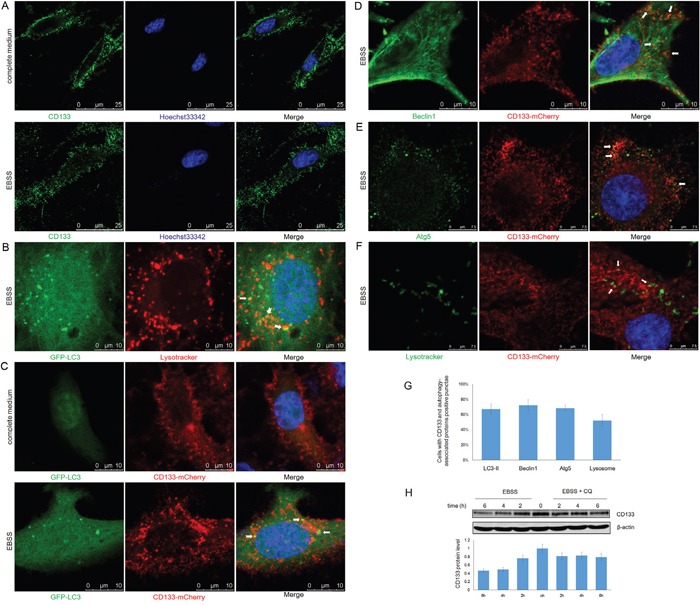
CD133 directly undergoes autophagosome formation and lysosome degradation **A.** C6-CD133 cells were fixed and stained for CD133 after culturing in complete medium or EBSS for 4h. Nuclei were visualized with Hoechst (blue). **B.** C6 cells were transfected with GFP-LC3 for 24h and were labeled with lysotracker (red) for 1h before observation. GFP-LC3 co-stained with lysotracker were determined by confocal microscope after culturing in EBSS for 4h. **C.** CD133-mCherry and GFP-LC3 were co-transfected into C6 cells. Cells were observed with confocal microscope in complete medium or EBSS for 4h, respectively. Representative CD133-mCherry and GFP-LC3 double-positive foci were indicated by arrowheads. **D.** C6 cells were transfected with CD133-mCherry for 24h and were stained by antibody for Beclin1. Localization of CD133 and Beclin1 were observed under confocal microscope in EBSS. **E.** C6 cells transiently expressing CD133-mCherry were stained for Atg5 by antibody after culturing in EBSS for 4h. CD133 and Beclin1 double-positive puncta were indicated by arrowheads. **F.** C6 cells transiently expressing CD133-mCherry were evaluated for CD133 and lysotracker double positive puncta in EBSS. Lysotracker (green) was added to cell medium for 1 h prior to observation. **G.** Quantification of cells displaying CD133 and autophagy-associated protein double positive punctae upon starvation shown in (C-F), at least 100 cells per experiment from three independent experiments were quantified. **H.** C6-CD133 cells were cultured under EBSS for indicated time with or without 50uM CQ, expression of CD133 was determined by Western blot. The folds of intensity were obtained by the ratios of the value of each group over control respectively and shown in graphs under immunoblots. Data of three replicates were shown as means ± SD.

CD133 inhibits the uptake of transferrin by clathrin and cholesterol-endocytosis processes [35]. The function of CD133 is altered by changes in its subcellular localization from the plasma membrane to the cytoplasm [36]. We further examined the role of CD133 in cell autophagy by analyzing changes to CD133 subcellular localization along with alterations to the tumor microenvironment. CD133 was largely localized to the cell membrane when cells were cultured in complete medium, while starvation reduced levels of membrane-associated CD133 and increased the cytoplasmic content of CD133 (Figure 5A). This suggests that CD133 was released from the membrane to the cytoplasm upon starvation. Consistent with the formation of autophagosomes that fuse with lysosomes to degrade proteins and organelles, LC3 exhibited partial co-localization with lysosomes labeled by lysotracker upon starvation (Figure 5B). Subsequently, C6 glioma cells were transiently co-transfected with CD133-mCherry and GFP-LC3 vectors to visualize the CD133 translocation following alterations to the microenvironment. CD133 was predominantly present on the cell membrane, with less co-localization with LC3 under normal culture conditions (Figure 5C). However, more and larger cytoplasmic vesicles formed after EBSS stimulation, Figure 5C depicts increased numbers of yellow particles representing CD133-mCherry co-localized with GFP-LC3 in the cytoplasm of C6 cells.

Partial localization of CD133 with either Beclin1 or Atg5 was identified by confocal fluorescence microscopy when cells were exposed to EBSS, indicating that a minute fraction of CD133 directly participates in autophagosomal membrane fusion (Figure 5D and 5E). Furthermore, starvation also induced the partial overlapping of lysotracker signal with CD133, indicating that CD133 ultimately undergoes degradation through lysosomes (Figure 5F). Consistently, Figure 5H demonstrates that CD133 levels were increased in the presence of CQ. Taken together, these findings demonstrate that CD133 directly undergoes autophagosome membrane fusion and lysosome degradation processes.

## DISCUSSION

When faced with insufficient nutrient availability, cancer cells can adapt by either tolerating starvation or finding ways to increase nutrient supply [37]. The specific tumor microenvironment controls cell behavior and leads to stress responses, including glycolytic metabolism alteration, apoptosis, and angiogenesis [38]. Low glucose or nutritional deprivation typically trigger autophagy. Increased expression of Atg proteins occurs in relation to autophagy induced by nutrient deprivation and oxygen deficiency [39]. While autophagy is important for the persistence of cancer cells upon starvation, the molecular mechanisms underlying this resistance still require further investigation. Our present study, demonstrates that CD133-expressing cells have a significantly increased survival capacity and undergo lower levels of apoptosis and necrosis following starvation. Inhibition of autophagy by CQ reduced the differences in levels of cell viability and apoptosis observed between populations of CD133^+^ and CD133^−^ cells. Additionally, activation of autophagy and autophagy-associated genes in glioma cells confers tolerance to nutritional starvation.

Recent studies have shown that mTOR inhibition can increase CD133^+^ subsets by abrogating differentiation of CD133^+^ cells and enhancing apoptosis of CD133^−^ subpopulations in liver cancer cell lines [40]. Rapamycin also up-regulates CD133 expression via inhibition of mTOR signaling in colorectal and gastric tumor cells [34]. However, limited data are available concerning the biochemical mechanisms by which CD133 influences cell regulation and protein-protein interactions within the autophagy and other signaling pathways in cancer cells. Our current study of CD133 expression following exposure of glioma cells to rapamycin differs from those previously conducted using colorectal and gastric cancer cells due to the different cellular context in glioma from the others, because CD133 expression in the C6-CD133 cell line was reproducibly and significantly downregulated by rapamycin. Inhibition of mTOR signaling by rapamycin reduced CD133 expression but up-regulated LC3-II levels. Therefore, CD133 may regulate a parallel signaling pathway that participates in crosstalk with mTOR signaling to regulate autophagy-associated genes. Furthermore, the influence of CD133 on autophagy can be eliminated by mTOR signaling.

CD133 is specifically localized to plasma membrane protrusions dependent on cholesterol-based membrane microdomains [15, 41]. Bauer et al. found that CD133 was released from the plasma membrane into culture media through the formation of exosomes which were then internalized by feeder cells during differentiation [19]. CD133-containing membrane particles are also released from neural progenitor cells into neural tube lumen during early phase of neurogenesis [42]. These results indicate that CD133 can respond to tumor microenvironment changes. Intriguingly, we identified a similar phenomenon in glioma cells, with effective autophagy occurring in response to stressful conditions. Additionally, partial co-localization of CD133 with LC3, Beclin1, Atg5, or lysosomes was detected, indicating that CD133 is associated with autophagosome maturation and lysosome degradation.

**Figure 6:**
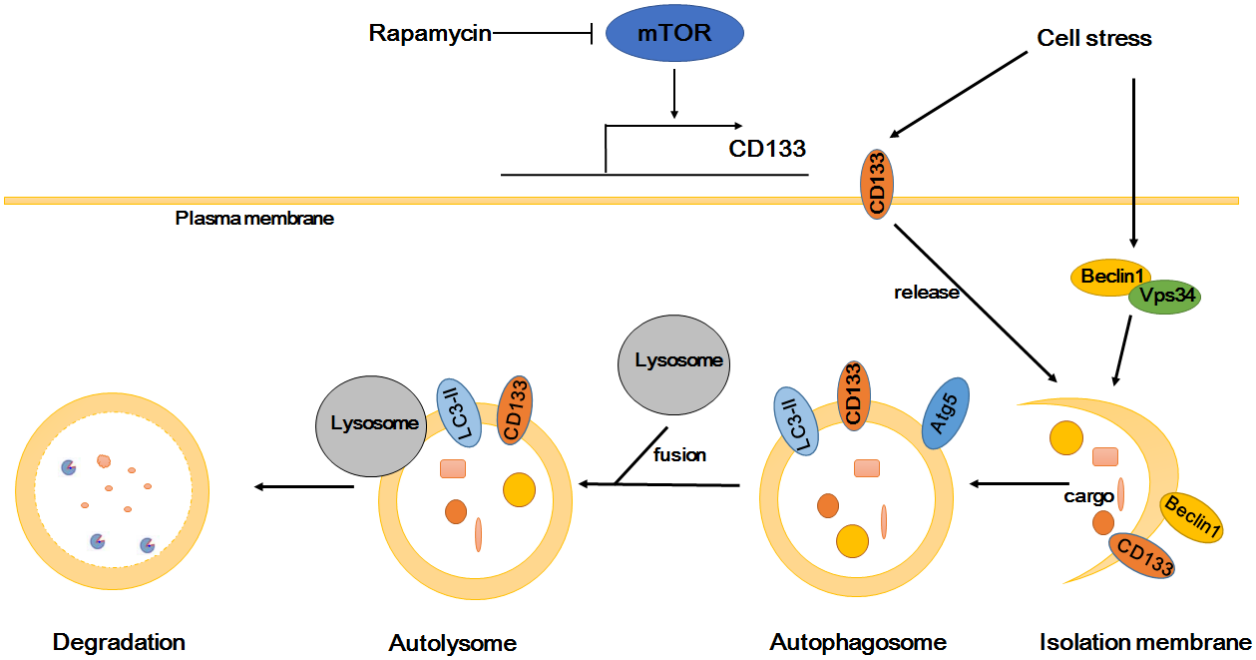
Proposed model depicting CD133 is upregulated by mTOR signal and participates in the autophagosome biogenesis CD133 expression can be regulated by mTOR signal. CD133 releases from membrane to cytoplasm upon starvation. Subsequently, CD133 directly participates in the membrane fusion process of autophagosome and ultimately fuses with lysosomes for degradation.

In conclusion, CD133 improves the resistance of glioma cells to a nutrient-deprived microenvironment by activating the autophagy-associated gene signaling pathway. The influence of CD133 on autophagy may be not as effective as mTOR signaling, and can be reversed by rapamycin. Considering previous studies alongside our results [43, 44], we propose that CD133 directly participates in autophagosome membrane fusion and lysosome degradation processes, with CD133-containing membrane particles potentially contributing to the membrane source of the phagophore (Figure 6). Our results provide a novel insight into the role of CD133 in cell regulation, and suggest that targeting CD133-linked signaling and autophagy in glioma cells may be helpful in improving anti-cancer treatments.

## MATERIALS AND METHODS

### Cell lines and reagents

F98 and C6 rat glioblastoma, human glioma U87 MG, and human embryonic kidney 293T cell lines were purchased from the American Type Culture Collection (Rockville, MD, USA), while the human glioma cell lines U251 and SHG44 were purchased from Institute of Basic Medical of Science, Research Chinese Academy of Medical Sciences. Cells were cultured in Dulbecco's modified Eagle's medium (DMEM) supplemented with 10% fetal bovine serum (HyClone, UT, USA) in a humidified atmosphere with 5% CO_2_ and 95% air at 37°C. Puromycin (Invitrogen, USA), rapamycin (Invitrogen), Chloroquine (CQ; Invitrogen) and 3-methyladenine (3-MA; Sigma–Aldrich, USA) were dissolved in DMSO prior to use.

### Plasmids construction and lentivirus transfection

The wild-type full-length human CD133 open reading frame was amplified from a cDNA template (ATCC number 10659084) by PCR and directionally cloned into pHBLV-CMVIE-IRES-Puro (a kind gift form Prof. Zebin Mao at Peking University Health Science Center) lentiviral vector at the *XbaI* and *NotI* sites. The PCR primers used are 5′-CGCATTTAAATATGGCCCTCGTACTCGG-3′ (forward) and 5′-GCCTTAATTAATCAATGTTGTGATGGGCTTGT-3′ (reverse). The recombinant construct was verified by DNA sequencing. GFP-LC3 and CD133-mCherry were constructed on pEGFP-C1 and pmCherry-N1 backbones, respectively. CD133 and Atg5 shRNA were inserted into the pSilencer2.0-u6 vector at the *BamHI* and *HindIII* sites. The sequences of shRNAs used are as follows: shCD133-1, GCTCCTAAGGCTTGGAATTAT; shCD133-2, GGACAAGGCGTTCACAGATCT; shCD133-3, GCTAGGAGGCGGAATTCTTGA; shAtg5-1, GACGGATTCCAACGTGCTTTA; shAtg5-2, GCATTATCCAATTGGCCTACT; shAtg5-3, GCAGTTGAGGCTCACTTTATG.

Glioma cells were transfected with pHBLV-CMVIE-IRES-Puro-CD133 vector according to previously described methods [30], followed by selection in DMEM medium containing puromycin for one week.

### Western blot analysis

Cells were collected and lysed in RIPA buffer (Beyotime, China) containing protease and phosphatase inhibitor cocktail (Roche) on ice for 30 min. Cell lysates were clarified by centrifugation at 4°C for 20 min. Total protein concentrations were measured using a Coomassie Protein Assay Kit (Pierce). Equal amounts of protein from each sample were separated on 10% or 15% SDS-PAGE gels and then electrotransferred to polyvinylidene fluoride membranes (Millipore). After blocking in 5% nonfat milk for 1 h at room temperature, the membranes were incubated overnight at 4°C with specified primary antibody against CD133/1 (AC133, Miltenyi), β-actin (M177-3, MBL), LAMP1 (ab13523, Abcam), LC3 (L7543, Sigma–Aldrich), P62 (ab56416, Abcam), Beclin1 (ab55878, Abcam), Atg5 (ab108327, Abcam), mTOR (ab32028, Abcam), p-mTOR (ab109268, Abcam), and caspase-3 (9662S, CST). After three washes with TBS containing 0.1% Tween-20, the membranes were probed with fluorescence-labeled anti-mouse or anti-rabbit secondary antibody (Rockland Immunochemicals) for 1 h at room temperature. Finally, the membranes were scanned using the Odyssey Fluorescent Western Scanning System (LI-COR, NE, USA). Fluorescence intensity was analyzed using ImageJ software.

### RNA isolation and quantitative real-time PCR

Total RNA was extracted using Trizol reagent (Invitrogen). cDNA was synthesized using the M-MLV reverse transcription kit (Promega, USA) following the manufacturer's instructions. PCR was performed with ExTaq (Takara, Japan). Quantitative PCR was performed using SYBR Green Real-time PCR Master Mix (Promega) and analyzed with the Mx3000P real-time PCR system (Agilent Technologies, CA, USA). The relative expression levels were normalized to those of GAPDH based on the Delta Ct method. Primers for real-time PCR are listed in Supplementary Table S1.

### Immunofluorescence microscopy

Cells were dissociated into single cell suspensions using trypsin and seeded onto cover slips in a 24-well plate. Cells were fixed by incubation with 4% paraformaldehyde for 15 min at room temperature after each experiment. Cells were permeabilized with 0.3% Triton X-100 for 15 min at room temperature and then blocked by incubation with 10% bovine serum albumin for 1 h at 37°C. Coverslips were then incubated at 4°C with primary antibody solution overnight, washed three times in PBS, and incubated with secondary antibody solution containing Hoechst 33342 for 1 h at 37°C. Secondary antibodies used were: Alexa Fluor®-488 and −594 goat anti-rabbit or anti-mouse IgG (ZSGB-BIO, China). Finally, coverslips were washed three times and mounted on microscope slides using mounting medium (ZSGB-BIO). Images were captured under a Leica TCS SP5 confocal laser-scanning microscope (Leica, IL, USA), with co-localization analysis and image merge conducted using Leica software according to the recommended procedures.

### Determination of cell viability

Cell Counting Kit-8 (CCK-8) assay (C0038, Dojindo Laboratories) was used to determine cell viability. F98 and C6 cells were firstly dissociated into single cell suspensions using trypsin and seeded into 96-well plate (1 × 10^4^ cells per well). At different time points, a mixture of 10 μl CCK-8 solution and 90 μl media was added to each well after the wells were rinsed with PBS. After incubation at 37°C for 1 h in a humidified CO_2_ incubator, absorbance was read at 450 nm by a microplate reader (VICTOR1420, PerkinElmer Life and Analytical Sciences). The values were used to calculate cell proliferation by setting the control as 100%.

### Autophagy assay

F98 and C6 Cells were transiently transfected with GFP-LC3 vector and then observed and imaged using a TCS SP5 confocal microscope after cultured under the indicated conditions. Five images were taken from each well, and cells with more than six green fluorescence puncta were classified as autophagic-positive cells. Cell numbers are expressed as means ± SD. A minimum of 1000 cells per sample were counted in triplicate for each experiment.

### Flow cytometry

The percentage of CD133-positive cells was determined using flow cytometry and the CD133/1(AC133)-PE (130-098-826, Miltenyi) antibody according to the manufacturer's instructions. Briefly, 1 × 10^6^ cells were collected following trypsinization and washed twice with ice-cold PBS. CD133/1(AC133)-PE and mouse IgG-PE isotype control (Miltenyi) antibodies were used at a 1:1000 dilution and incubated with cells for 30 min on ice in the dark. Cells were then washed twice with ice-cold PBS and analyzed using a FACSCalibur flow cytometer (BD Biosciences, NJ, USA).

### Cell apoptosis assay

An Annexin V-FITC and propidium iodide (PI) double staining assay was used to measure apoptotic and necrotic cells by flow cytometry according to the manufacturer's instructions (C1062, Beyotime). Briefly, 1 × 10^6^ cells were collected by trypsinization, washed twice with ice-cold PBS, and resuspended in 200 μl binding buffer containing 10 μl Annexin V and 5 μl PI. After incubation for 30 min on ice in the dark, cells were analyzed using a FACSCalibur flow cytometer.

### Statistical analysis

All experiments were repeated at least three times. The data are expressed as means ± SD. Statistical analysis of two experimental groups was performed using two-tailed Student's *t*-tests. One-way ANOVA was performed to compare more than two groups. *P*-values less than 0.05 were considered significant.

## SUPPLEMENTARY FIGURES AND TABLE


