# Supplementary material for: Resistance of glioma cells to nutrient-deprived microenvironment can be enhanced by CD133-mediated autophagy
Source: Oncotarget. 2016 Oct 21;7(46):76238–49. doi: 10.18632/oncotarget.12803 (PMC5342810; doi:10.18632/oncotarget.12803)
Supplement: Supplementary file 1 [file oncotarget-07-76238-s001.pdf]

Resistance of glioma cells to nutrient-deprived microenvironment can be enhanced by CD133-mediated autophagy

SUPPLEMENTARY FIGURES AND TABLE

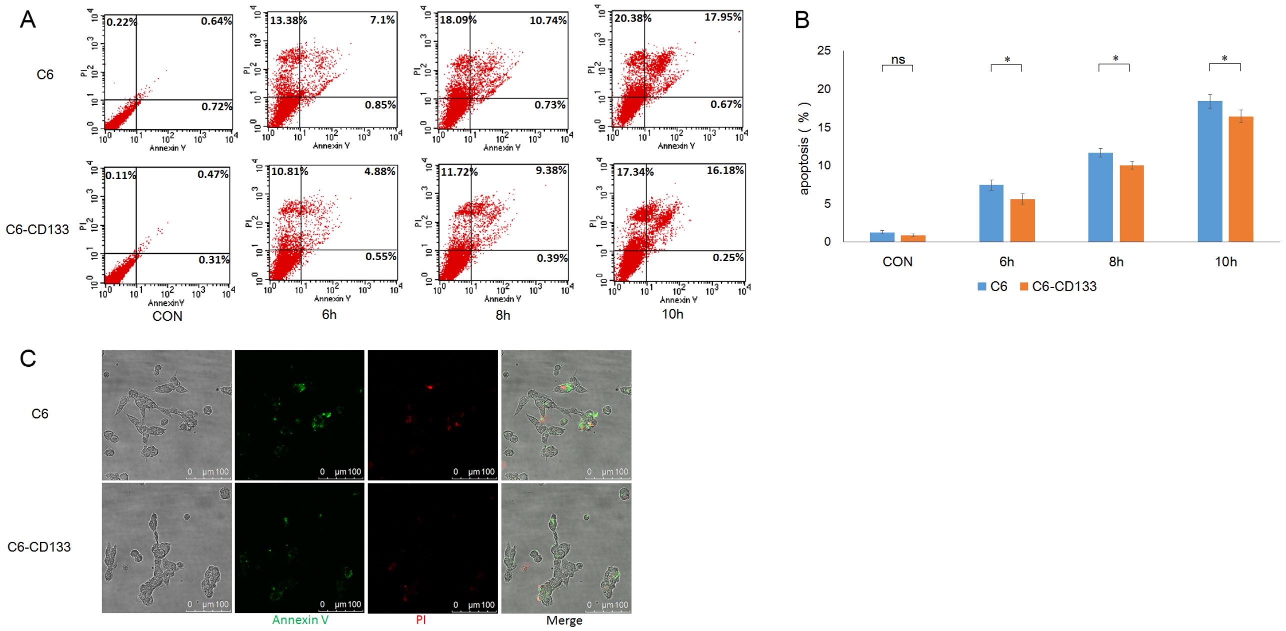

Supplementary Figure S1: A. C6 and C6-CD133 cells were treated with EBSS for 6, 8 and 10h to determine the apoptosis and necrosis. The percentage of apoptosis and necrosis was analyzed by Annexin V-FITC/PI double staining via flow cytometry after the designated treatments. **B.** Percentage of Annexin V positive cells was quantified. **P*<0.05. **C.** The percentage of apoptosis and necrosis was evaluated by Annexin V-FITC/PI double staining via immunofluorescence microscopy after C6/C6-CD133 cells exposed to EBSS for 10h.

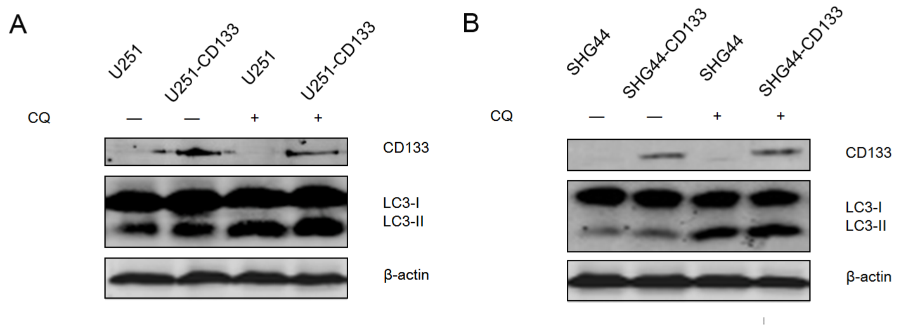

Supplementary Figure S2: A, B. Levels of LC3-II in these indicated cell lines were evaluated by Western blot after incubating in EBSS for 4h with or without 50uM CQ.

Supplementary Table S1: Primers used for PCR

Gene	Forward / Reverse primer	Primer Sequence (5'-3')
CD133	F	AGTGGCATCGTGCAAACCTG
	R	CTCCGAATCCATTCGACGATAGTA
Nanog	F	CTAAGAGGTGGCAGAAAAACA
	R	CTGGTGGTAGGAAGAGTAAAGG
Sox2	F	CATGTCCCAGCACTACCAGA
	R	GTCATTTGCTGTGGGTGATG
Oct4	F	GTACTCCTCGGTCCCTTTCC
	R	CAAAAACCCTGGCACAAACT
c-Myc	F	GCCACGTCTCCACACATCAG
	R	TGGTGCATTTTCGGTTGTTG
GAPDH	F	GGACCTGACCTGCCGTCTAGAA
	R	GGTGTCGCTGTTGAAGTCAGAG
